# Physicians and Druggists

**Published:** 1880-11

**Authors:** 


### Editorial.

### PHYSICIANS AND DRUGGISTS.

The relation between apothecaries and physicians is so intimate and their dependence one upon the other so perfect, that a proper understanding of the duties of each is all important to the public. Differences of opinion in this particular disturb that confidence and respect for each other which should exist; and lead to embarrassment and confusion amongst their patrons, whose interest both parties are expected to protect. Practitioners of medicine and prescriptionists should agree upon a code of pharmaco-medical ethics. It is their mutual interest to do so, as well as that of the public. With bickerings, misunderstanding and jealousies between physicians and druggists the public have no safety, and the sooner they discard such professional men the better for invalids.

The rights of patients seem to be entirely overlooked in the contention between the professions. Physicians complain of the alleged improper use of their prescriptions by prescriptionists, while the latter and the patient declare the restriction unjust and injurious to themselves. The question to be settled is, what right the patient has in the prescription? Is it his to do with it as he may think proper, or does the prescriber retain a right to it after being filled and filed in the apothecary's shop? The right of apothecaries to fill prescriptions written by persons other than physicians, must be determined in order to the proper solution of this question. If such right be acknowledged, then the refilling of physician's prescriptions is unavoidable, because copies without the physician's name can be made to any number desired before having it filled. So, it will be observed that the whole difficulty can be settled by a decision upon this question. Let us come squarely to the point and settle the matter finally by an agreement on this question. Can a receipe for pills, made with equal parts of aloes and rhubarb, without the name of a known physician, be legitimately filled by a prescriptionist? If so, the question is settled that prescriptions can be properly refilled without the writer's consent; for any prescription can be prescribed in the same way and as often as the holder may desire. If not, they could not be allowed to sell an ounce of castor oil and ten drops of turpentine, nor even an ounce of epsom salt. If physicians would retain a right to their prescriptions, they must necessarily retain the possession until they deliver them in person to the apothecary for filling and filing. This no physician would consent to do, and, therefore, could not expect the apothecary to know who wrote the original of a copy, or whether it had been filled twenty times before or not at all. This trouble must be settled between the physician and patient, and if the former cannot trust the patient to deal fairly with him, he should not require the utter impossibility of doing so by the prescriptionist.

We cannot, therefore, see how physicians can, with propriety, hold druggists to a strict accountability for the refilling of prescriptions, though the patient should have no right to use them the second time. As above stated, we think the quarrel should be between patient and physician, if a quarrel must be had. We must not be understood as holding that refilling prescriptions without the physician's directions, is proper, beneficial or safe to the patient. No; we think it is a wrong and unsafe practice, but the damage accrues to the patient more than to the physician. It is true, the former may lose occasionally the fee for writing a new prescription, but if the patient should expect to save money by such means, he will find he is mistaken; for the use of an old prescription at a time when the condition and circumstances have changed, may not only fail to benefit him, but often lead to a worse state, which will require additional prescriptions and visits, making his pecuniary outlay and the physician's gain greatly more than if he had pursued the proper course of getting his physician's advice instead of taking the treatment in his own hands.

There is, however, a practice in which some prescriptionists indulge, that should be condemned by all friends of humanity, viz: the practice of prescribing for patients. While physicians, perhaps, lose nothing in the end from such habit, yet, it becomes them as conservators of the public interest, to reprove and condemn in the strongest terms a course like this, which puts money in the druggist's pocket at the personal risk of citizens. Apothecaries are not physicians, and are not sufficiently familiar with the nature of disease and the action of medicines to prescribe beneficially or even safely for cases of disease. Even educated and successful practitioners, who lay aside the practice for the active duties of an apothecary, soon lose the capacity of performing the duties of a physician. One of the professions is entirely sufficient for one man, and when both are attempted by one man, the public suffer, and he himself is not pecuniarily benefitted. It is not from the danger of his own pecuniary loss that the physician should complain of this practice, but, as a conservator of the public weal, he should set his face against every strategem of designing men to secure pecuniary gain by deceiving invalids into the erroneous belief that remedies can be successfully distributed without the proper, or even any qualifications at all.

This brings us to the consideration of the monster evil of the land, which is generally fostered and encouraged by druggists, for their own pecuniary benefit, but greatly to the bodily and pecuniary injury of the people of this country. Secret nostrum vending is certainly the greatest evil connected in any way with medicine. Physicians generally condemn the practice when interrogated on the subject, and adopt strict rules of ethics for the government of themselves on this subject, but the public are made to believe by the nostrum maker and vender, that all this flummery of physicians is had to prevent speedy cures by any other than themselves. So that what we say to the public on the subject is made the means of increasing the sale of secret remedies and making millionaires of advertising inventors.

There is one plan, however, by which physicians can compel a great reduction of this traffic. Druggists are dependent on physicians, and if the latter should require his apothecary to sell nothing but legitimate medicines or lose his patronage, a practical move in the right direction will have been made. If we are sincere in our profession of opposition to the sale of secret nostrums, we certainly will adopt a course which will effect the object. Druggists themselves, and physicians apologizing for them, say that it is impossible to keep up a drug store without keeping secret nostrums; that the people demand them, and that the druggist who attempts the drug business without them must fail. Now, this has been proven to be an error. Even without an equal proportion of physicians' patronage, a prescription store has been in successful operation in the city of Atlanta more than a year, from which secret nostrums are entirely excluded.

Whether in failing to give this store an equal proportion of the patronage physicians expect more influence in their business from other druggists, or are really in favor of that which the ethics of their profession compel them nominally to condemn, we cannot determine. The facts, however, exist, and the drug store without secret remedies owes no one, has money on hand, and will not fail.

